# Impact of HER2-targeted PET/CT imaging in patients with breast cancer and therapeutic response monitoring

**DOI:** 10.1093/oncolo/oyae188

**Published:** 2024-07-31

**Authors:** Xinyu Gui, Xu Liang, Xiaoyi Guo, Zhi Yang, Guohong Song

**Affiliations:** Key Laboratory of Carcinogenesis and Translational Research (Ministry of Education/Beijing), Department of Breast Oncology, Peking University Cancer Hospital and Institute, Beijing 100142, People’s Republic of China; Key Laboratory of Carcinogenesis and Translational Research (Ministry of Education/Beijing), Department of Breast Oncology, Peking University Cancer Hospital and Institute, Beijing 100142, People’s Republic of China; Key Laboratory of Carcinogenesis and Translational Research (Ministry of Education/Beijing), NMPA Key Laboratory for Research and Evaluation of Radiopharmaceuticals (National Medical Products Administration), Department of Nuclear Medicine, Peking University Cancer Hospital & Institute, Beijing 100142, People’s Republic of China; Key Laboratory of Carcinogenesis and Translational Research (Ministry of Education/Beijing), NMPA Key Laboratory for Research and Evaluation of Radiopharmaceuticals (National Medical Products Administration), Department of Nuclear Medicine, Peking University Cancer Hospital & Institute, Beijing 100142, People’s Republic of China; Key Laboratory of Carcinogenesis and Translational Research (Ministry of Education/Beijing), Department of Breast Oncology, Peking University Cancer Hospital and Institute, Beijing 100142, People’s Republic of China

**Keywords:** breast cancer, HER2-targeted PET/CT, response monitor, tumor heterogeneity

## Abstract

**Background:**

Patients with breast cancer exhibit heterogeneity in the expression of the human epithelial growth factor receptor 2 (HER2). Clinically, re-biopsying recurrent or metastatic lesions presents substantial challenges. This study aimed to evaluate the efficacy of HER2-targeted PET/CT imaging in identifying HER2 expression in breast cancer lesions and monitoring therapeutic responses.

**Patients and Methods:**

This exploratory analysis used data from a prospective study that included adult patients with breast cancer who underwent both Al^18^F-NOTA-HER2-BCH and ^18^F-FDG PET/CT imaging at Beijing Cancer Hospital between June 2020 and July 2023 (NCT04547309).

**Results:**

Fifty-nine participants, with a median age of 55 years, were analyzed. Lesions imaged with HER2-targeted PET/CT before anti-HER2 therapy exhibited higher SUVmax values than after therapy in HER2 immunohistochemistry (IHC) 3 + lesions (19.9, 95% CI: 15.7-25.3 vs 9.8, 95% CI: 5.6-14.7; *P* = .006). A significant positive correlation was observed between SUVmax on HER2-targeted PET/CT and IHC before therapy (*P* = .034), with higher SUVmax values noted in lesions with positive HER2 pathology compared to those with negative HER2 status (17.9 ± 13.2 vs 1.1 ± 0.3; *P* = .007). HER2 expression heterogeneity was confirmed both between primary and metastatic lesions (22.9%) and among different metastatic sites (26.7%) as assessed by HER2-targeted PET/CT. A superior therapeutic response correlated with higher pretreatment SUVmax values. The HER2-targeted PET/CT procedure was well-tolerated by all patients.

**Conclusion:**

HER2-targeted PET/CT imaging offers a practical, non-invasive, and quantitative approach for assessing HER2 status in breast cancer patients, facilitating the optimization and personalization of therapeutic strategies by oncologists.

## Teaser

This study evaluated the efficacy of HER2-targeted PET/CT imaging in identifying HER2 expression in breast cancer lesions and monitoring therapeutic responses.

## Graphic Abstract

**Figure:**
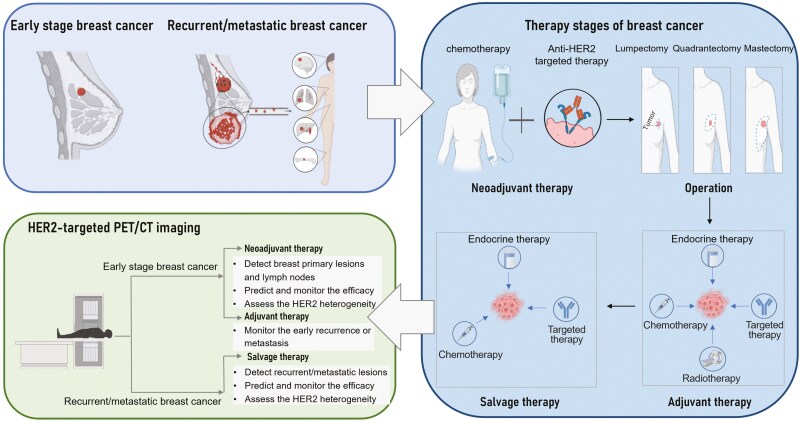

**Implications for practice**

HER2-targeted PET/CT imaging can detect the HER2 expression of various lesions in patients with breast cancer in a real-time, noninvasive, and quantitative manner, providing an effective solution to problems including the difficulty of re-biopsy, the existence of tumor heterogeneity, and the lack of comprehensive efficacy evaluation in clinical practice. This imaging approach has the potential to help oncologists provide optimized and individualized therapy for patients with breast cancer.

## Introduction

The prognostic outcomes for patients with breast cancer, categorized by molecular types and stages, exhibit significant variance, with an estimated 20% to 30% experiencing recurrence and metastasis following radical mastectomy.^1,2^ Approximately 15% to 20% of cases are characterized by the positivity of the human epithelial growth factor receptor 2 (HER2), a subset associated with comparatively adverse prognoses and diminished overall survival.^3,4^ The implementation of anti-HER2 therapy throughout the disease is advocated to optimize survival outcomes in patients with advanced HER2-positive breast cancer. Notably, HER2 expression within breast cancer demonstrates up to 34% heterogeneity, potentially manifesting as spatial intralesional variance (within a singular lesion), spatial interlesional disparity (among primary and metastatic lesions, as well as among distinct metastases), and temporal heterogeneity across treatments.^5-8^ This heterogeneity in HER2 may serve as an unfavorable predictor for the efficacy of HER2-targeted treatments in HER2-positive breast cancer.^8^ Consequently, accurate determination of HER2 expression is imperative for formulating treatment approaches for advanced breast cancer, with guidelines universally recommending for the repeated biopsy of metastatic sites to elucidate the molecular subtype.^2-4^ Recent studies have illuminated the dynamic status of HER2-low among patients with triple-negative breast cancer (TNBC) through serial biopsies, revealing an increased likelihood of a HER2-low result correlating with the number of biopsy procedures.^9^ Nonetheless, the practical challenges and inherent risks associated with multiple and multisite biopsies are pronounced. Patients lacking metastatic biopsy data are conventionally treated based on the molecular subtype of the primary lesion, a practice potentially misguided by tumor heterogeneity.

The utility of positron emission tomography/computed tomography (PET/CT) molecular imaging in breast cancer encompasses a broad spectrum of applications, including lesion detection, tumor staging, assessment of heterogeneity, evaluation of treatment efficacy, and ongoing surveillance, in addition to its role in facilitating informed treatment decisions.^10-12^ This modality also extends to the evaluation of estrogen receptor (ER)/progesterone receptor (PR) and HER2 status, thereby enabling a comprehensive analysis of the molecular subtype of both primary and metastatic lesions.^4^ The traditional PET/CT imaging agent, ^18^F-fluoro-2-deoxyglucose (^18^F-FDG), reflects the metabolic activity of tumor cells through its accumulation, although it exhibits reduced sensitivity toward lesions within the brain and liver.^13^ Conversely, HER2-targeted molecular probes are designed to specifically bind to HER2 receptors on tumor cell surfaces, thus permitting an accurate determination of HER2 expression across various systemic lesions.^14-17^ The capacity for dynamic monitoring of HER2 expression via this modality offers invaluable insights for the optimization of anti-HER2-targeted therapy. Consequently, HER2-targeted PET/CT imaging, by virtue of its non-invasive and quantitative nature, emerges as an efficacious approach to address the clinical challenges associated with biopsy procedures and the heterogeneity of HER2 expression.^18,19^

Leveraging the resources of the Department of Nuclear Medicine at Beijing Cancer Hospital, our research team has engaged in the investigation and clinical translation of Al^18^F-NOTA-HER2-BCH, implementing HER2-targeted PET/CT imaging in HER2-positive patients. This effort has culminated in a substantial compilation of clinical case studies and a solid research foundation.^20,21^ The present study used the Al^18^F-NOTA-HER2-BCH agent to evaluate its safety, feasibility, biodistribution, and efficacy in targeting tumors within breast cancer patients. This study aimed to elucidate the clinical utility of HER2-targeted PET/CT imaging for patients with breast cancer, with a particular focus on dissecting the correlation between PET/CT outcomes and response evaluation. This encompasses examining the relationship between maximum standardized uptake values (SUVmax) obtained through HER2-targeted PET/CT imaging and HER2 immunohistochemistry (IHC) results, as well as the association between SUVmax and tumor response amidst tumor heterogeneity. The findings are anticipated to furnish novel theoretical insights and clinical guidelines for refining anti-HER2 therapeutic strategies.

## Methods

### Study design and patients

This was an exploratory analysis derived from a prospective study conducted at the Beijing Cancer Hospital. The study spanned from June 2020 to July 2023, focusing on patients who underwent both HER2-targeted and ^18^F-FDG PET/CT imaging. The objective of the prospective study was to assess the comparative efficacy of Al^18^F-NOTA-HER2-BCH and ^18^F-FDG in identifying HER2-positive breast cancer lesions via PET/CT imaging. Ethical approval for this study was granted by the Medical Ethics Committee of Beijing Cancer Hospital (Ethics Approval License No. 2019KT114), and the study was registered on the Clinical Trial registry website (https://register.clinicaltrials.gov, NCT04547309). Informed consent was obtained from the participants included in this study. Analysis of medical records and PET/CT imaging outcomes was undertaken by 2 board-certified oncologists.

The inclusion criteria were (1) adults (aged 18 years and above) diagnosed with breast cancer (either early or advanced stage) through pathological biopsy and IHC staining; (2) documentation of HER2 status confirmed pathologically; (3) the presence of measurable or evaluable disease as per the Response Evaluation Criteria in Solid Tumors (RECIST) version 1.1^22^; (4) an Eastern Cooperative Oncology Group (ECOG) performance status score of ≤2, coupled with a life expectancy of more than 3 months; (5) consent to participate in both HER2-targeted and ^18^F-FDG PET/CT imaging studies at Beijing Cancer Hospital from June 2020 to July 2023. The exclusion criteria were (1) patients with other primary malignant tumors, congestive heart failure, or severe hepatic or renal dysfunction; (2) receipt of PET/CT imaging during the adjuvant therapy setting; (3) pregnancy or lactation; (4) inability to remain supine on the scanner bed for a duration exceeding 1 hour.

All participants had at least one biopsy-proven lesion for HER2 status assessment, predominantly sourced from breast lesions. In cases of metastatic breast cancer with multiple distant metastases, biopsy verification was limited to a minority of lesions due to ethical considerations. HER2 positivity was determined in accordance with American Society of Clinical Oncology (ASCO)/College of American Pathologists (CAP) guidelines, defined by an IHC score of 3+, or an IHC score of 2 + with HER2 amplification confirmed by fluorescence in situ hybridization (FISH). A HER2-low status was identified by an IHC score of 1+, or an IHC score of 2 + without HER2 amplification by FISH. Hormone receptor (HR) positivity was defined as an ER and/or PR expression level of ≥1% in either the primary or metastatic lesions.

### HER2-targeted and ^18^F-FDG PET/CT imaging

The imaging agent Al^18^F-NOTA-HER2-BCH was synthesized at Beijing Cancer Hospital and prepared for administration to the study participants by technicians. Each participant received an injection of 222 ± 18.5 MBq of Al^18^F-NOTA-HER2-BCH, followed by imaging at 2 hours post-injection. To mitigate nonspecific hepatic uptake, a co-injection of 1 mg of unlabeled HER2-affibody was administered, as outlined in our prior study.^23^ Subsequent to this, all subjects underwent an ^18^F-FDG PET/CT scan, administered at a dose of 3.7 MBq/kg, 1-hour post-injection, ensuring a minimum fasting period of 6 hours prior to the procedure, within a 7-day window.

Imaging was conducted using a Biograph mCT Flow 64 scanner (Siemens, Erlangen, Germany) ranging from the apex of the skull to the mid-thigh. The PET axial field of view was set at 21.6 cm, with PET data acquisition in a 3-dimensional mode utilizing flow motion technology (bed entry speed of 1 mm/s). Image reconstruction was performed utilizing the Siemens Company Multimodality Workplace (MMWP) platform, employing the TrueX + TOF algorithm. Attenuation correction was facilitated by unenhanced low-dose CT data.

### Image analysis

Image interpretation was conducted by 2 experienced radiologists. The uptake of the tracer within lesions was quantified utilizing the SUVmax, with the quantification of SUVmax facilitated by the automatic delineation of the 3-dimensional volume encompassing a 60% region of interest (ROI). A lesion identified on PET/CT imaging was classified as suspiciously positive if its SUVmax exceeded that of the adjacent tissues. For the semiquantitative assessment of PET/CT scans, any localized accumulation of ^18^F-FDG and/or Al^18^F-NOTA-HER2-BCH surpassing adjacent tissue levels was regarded as indicative of a positive focus, suggestive of a potentially malignant lesion.

### Evaluation of efficacy

The evaluation of tumor response used the RECIST version 1.1, utilizing radiological imaging techniques (CT or magnetic resonance imaging [MRI]). Response categorization included complete response (CR), partial response (PR), stable disease (SD), and progressive disease (PD). The objective response rate (ORR) represented the percentage of patients experiencing either a CR or PR. Progression-free survival (PFS) was defined as the duration from the commencement of an antitumor treatment regimen to the date of disease progression or death, whichever came first. Patient monitoring was conducted every 2 cycles during antitumor therapy, including regular physical examinations, hematologic and serum biochemical evaluations, and imaging assessments through CT or MRI scans.

### Statistical analysis

The dataset including demographics, clinical outcomes, and PET/CT imaging data was derived from patient medical records and subjected to descriptive analysis. Categorical variables were presented as counts (percentages). Group comparisons were conducted utilizing the chi-square test or the Mann-Whitney *U* test, as deemed applicable. The association between PET/CT-derived SUVmax values and HER2 IHC scores was examined using Pearson’s correlation coefficient. PFS estimates by Kaplan-Meier analysis were evaluated for differences employing the log-rank test. Statistical analyses were performed using SPSS software, version 15.0 (SPSS Inc., Chicago, IL), and a *P* value of < 0.05 was considered statistically significant.

## Results

### Patient characteristics

From June 2020 to July 2023, 86 breast cancer patients underwent both Al^18^F-NOTA-HER2-BCH PET/CT and ^18^F-FDG PET/CT imaging. Following the application of inclusion and exclusion criteria, 59 female patients were included in this study. The median age of participants was 55 years (range: 30-88 years). Of these, 50 patients (84.7%) were HER2 positive, whereas 9 patients (15.3%) were HER2 negative. Fifteen patients (25.4%) received PET/CT imaging for initial tumor evaluation either before (9/15, 60.0%) or shortly after (6/15, 40.0%) commencing neoadjuvant therapy. Conversely, 44 patients (74.6%) underwent PET/CT imaging following recurrence or metastasis, either before (13/44, 29.5%) or after (31/44, 70.5%) initiation of first-line therapy. A summary of patients’ demographic information and baseline clinical characteristics is provided in Table 1. The HER2-targeted PET/CT procedure was well tolerated by all patients, with no observed adverse pharmacological effects or physiological responses attributed to Al^18^F-NOTA-HER2-BCH administration.

**Table 1.** Demographic and baseline clinical characteristics of female patients with breast cancer (*n* = 59).

Characteristics	*n* (%)
Age, years [median (range)]	55 (30-88)
ECOG performance status score	
0	44 (74.6)
1	14 (23.7)
2	1 (1.7)
Histologic type	
Invasive ductal carcinoma	50 (84.7)
Invasive lobular carcinoma	7 (11.9)
Micro papillary carcinoma	1 (1.7)
Metaplastic carcinoma	1 (1.7)
HR status	
HR+	20 (33.9)
HR–	39 (66.1)
HER2 status	
HER2 positive	50 (84.7)
HER2 negative	9 (15.3)
Current phase of treatment for breast cancer	
Neoadjuvant therapy	15 (25.4)
Salvage treatment	44 (74.6)
Metastatic sites in patients (*n* = 44)	
Bone	24 (54.5)
Lung	17 (38.6)
Liver	12 (27.3)
Breast wall	11 (25.0)
Brain	3 (6.8)

Abbreviations: ECOG, Eastern Cooperative Oncology Group; HR, hormone receptor; HER2, human epidermal growth factor receptor-2; TNBC, triple negative breast cancer.

### SUVmax in HER2-targeted PET/CT imaging and HER2 IHC

**Figure 1.**
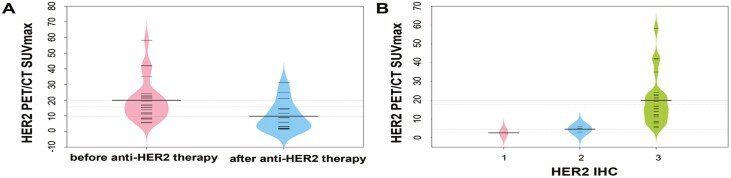
(A) Among the 39 lesions with HER2 IHC 3+, the SUVmax of lesions before anti-HER2 therapy was compared with that of lesions after anti-HER2 therapy; (B) The correlation between the SUVmax in HER2-targeted PET/CT and HER2 immunohistochemistry (IHC) status in the 27 patients who had received HER2-targeted PET/CT imaging before anti-tumor therapy.

In our study, 48 lesions identified in patients were evaluated through both pathological IHC and HER2-targeted PET/CT imaging. Of these, 27 lesions (56.3%) underwent HER2-targeted PET/CT imaging prior to the initiation of anti-tumor therapy, while the remaining 21 lesions (43.8%) were imaged during anti-tumor therapy. Within this subset, 39 lesions demonstrated a HER2 IHC score of 3+, with 23 patients receiving HER2-targeted PET/CT before anti-HER2 therapy and 16 patients undergoing imaging post-therapy commencement. Notably, the SUVmax values for lesions prior to anti-HER2 treatment were significantly higher than those observed following treatment (19.9, 95% CI: 15.7-25.3 vs 9.8, 95% CI: 5.6-14.7; *P* = .006; Figure 1A).

Given the impact of anti-HER2 therapy on reducing SUVmax in lesions, we further explored the relationship between SUVmax and HER2 status (determined by IHC and FISH) in 27 patients imaged before anti-tumor therapy. This analysis revealed a significant correlation (*P* = .034; Figure 1B), with an average SUVmax of 19.9 in the 23 patients who were HER2 IHC 3 + prior to therapy. Lesions with positive HER2 pathology (24 out of 27, 88.9%) exhibited higher SUVmax values compared to those with negative HER2 pathology (3 out of 27, 11.1%) (17.9 ± 13.2 vs 1.1 ± 0.3; *P* = 0.007).

**Figure 2.**
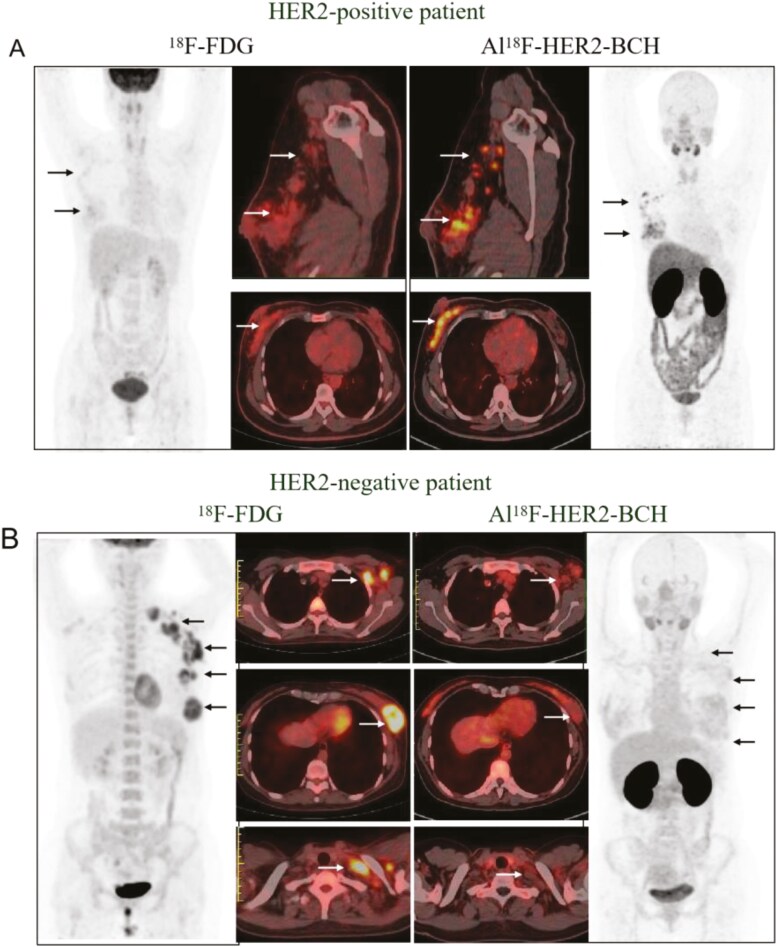
Images of ^18^F-FDG PET/CT and Al^18^F-NOTA-HER2-BCH PET/CT in a HER2-positive and HER2-negative patient. (A) A 47-year-old patient with primary diffuse breast cancer and multiple lymph nodes. Biopsy proved IHC 3+. (B) A 39-year-old with recurrence and metastasis. Biopsy proved IHC 1+.

As depicted in Figure 2A, Al^18^F-NOTA-HER2-BCH was superior in detecting primary and suspected lymph node lesions compared to ^18^F-FDG (20 vs 7 lesions). The average diameter of axillary lymph nodes detected was 0.72 cm, with the smallest measuring only 0.29 cm. Additionally, Al^18^F-HER2-BCH identified 6 lymph nodes smaller than 0.50 cm. In the context of HER2-negative lesions (Figure 2B), Al^18^F-NOTA-HER2-BCH exhibited lower uptake in tumor sites, contrasting with high uptake observed with ^18^F-FDG, aligning with the low HER2 expression determined by IHC.

### Tumor heterogeneity

The heterogeneity of HER2 expression in breast cancer patients was evident, including both primary and metastatic sites as well as among various metastatic lesions. Histopathological assessment and IHC methods were used to evaluate the primary lesions in all 59 patients with breast cancer, revealing HER2 positivity in 43 of the 59 patients (72.9%) and HER2 negativity in 16 of the 59 patients (27.1%). Metastatic lesions in 35 patients (59.3%) underwent biopsy, of which 28 (80.0%) were found to be HER2 positive and 7 (20.0%) were HER2 negative. Of the 35 patients who underwent both primary and metastatic lesion evaluation, 21 (60.0%) exhibited HER2 positivity in both sites, 6 (17.1%) were HER2 negative in both, and 8 (22.9%) displayed HER2 expression heterogeneity between the primary and metastatic sites. The HER2 expression data for the 8 patients with notable heterogeneity are detailed in Table 2.

**Table 2.** The pathological HER2 expression of the 8 patients with breast cancer who had heterogeneity between the primary and metastatic focus

No.	HER2 IHC of primary focus	HER2 FISH of primary focus	HER2 status of primary focus	HER2 IHC of metastatic focus	HER2 FISH of metastatic focus	HER2 status of metastatic focus	HER2 PET/CT SUVmax in recurrent stage
1	0	NA	Negative	2+	Positive	Positive	1.8
2	0	NA	Negative	3+	NA	Positive	10.9
3	2+	Negative	Negative	2+	Positive	Positive	7.3
4	2+	Negative	Negative	3+	NA	Positive	8.7
5	0	NA	Negative	3+	NA	Positive	8.7
6	2+	Positive	Positive	1+	NA	Negative	8.5
7	1+	Negative	Negative	3+	NA	Positive	35.7
8	1+	Negative	Negative	2+	Positive	Positive	2.4

Abbreviations: HER2, human epidermal growth factor receptor-2; IHC, immunohistochemistry; FISH, fluorescence in-situ hybridization; SUV, standard uptake value; NA, not available.

**Figure 3.**
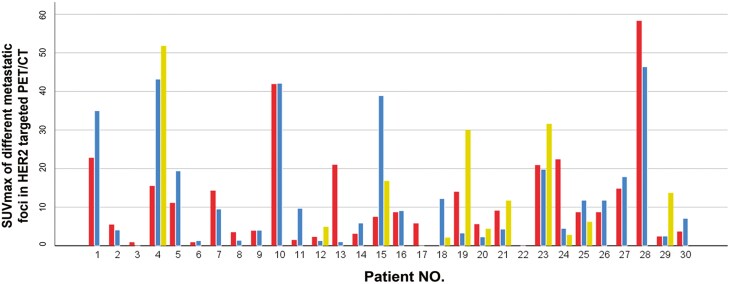
The SUVmax of different metastatic foci in HER2-targeted PET/CT among the 30 patients with advanced breast cancer.

In the cohort subjected to HER2-targeted PET/CT imaging, more than one lesion was assessed in 30 patients. Through semiquantitative analysis, consistent HER2 expression levels were identified in 22 of the 30 patients (73.3%), whereas 8 patients (26.7%) demonstrated variability in HER2 expression levels. Furthermore, the SUVmax values of various metastatic lesions in these 30 patients underscored the visible heterogeneity in HER2 expression by HER2-targeted PET/CT imaging (Figure 3).

### SUVmax of HER2-targeted PET/CT imaging and tumor response

**Figure 4.**
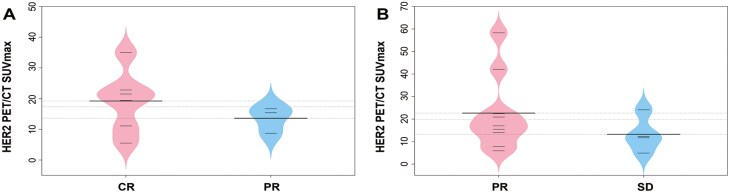
(A) The lesion SUVmax of HER2-targeted PET/CT in the patients with CR (*n* = 6) and PR (*n* = 3) in the 9 patients with HER2-positive breast cancer who had received HER2-targeted PET/CT imaging before neoadjuvant therapy; (B) The lesion SUVmax of HER2-targeted PET/CT in the patients with PR (*n* = 9) and SD (*n* = 4) in the 13 patients with HER2-positive breast cancer who had received HER2-targeted PET/CT imaging before the first-line therapy.

Among 9 patients with HER2-positive breast cancer undergoing HER2-targeted PET/CT imaging prior to neoadjuvant therapy, a pathological complete response (pCR) was observed in 66.7% (6/9) of the cases, with the remaining 3 patients demonstrating a PR upon pre-surgical evaluation. While the SUVmax values in HER2-targeted PET/CT imaging for patients achieving pCR exhibited a higher trend compared to those with PR, this difference did not reach statistical significance (*P* = .302; Figure 4A). Of these 9 patients, eight (88.9%), including 6 pCR and 2 non-pCR respondents, proceeded with adjuvant therapy with trastuzumab and pertuzumab, whereas one patient (11.1%) with non-pCR received adjuvant treatment with trastuzumab emtansine (T-DM1) post-surgery.

Furthermore, the PFS and best overall response were assessed in 13 patients with HER2-positive advanced breast cancer who had undergone HER2-targeted PET/CT imaging before initiating first-line therapy. The median PFS had not been reached at the time of analysis, with a response of PR in 9 out of 13 patients (69.2%) and SD in 4 out of 13 patients (30.8%). Although patients with PR appeared to exhibit higher SUVmax values in HER2-targeted PET/CT than those with SD, this difference was not statistically significant (*P* = 0.280; Figure 4B). No significant correlation was identified between SUVmax in HER2-targeted PET/CT and PFS following first-line treatment among patients experiencing PD, with a Pearson’s correlation coefficient of 0.092. After first-line therapy failure, 2 patients transitioned to second-line treatment with pyrotinib plus capecitabine, while another 2 were administered T-DM1.

**Figure 5.**
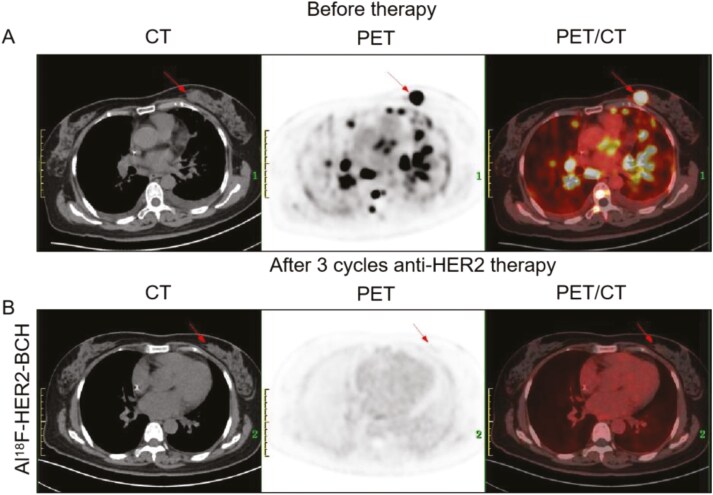
^18^F-FDG (A) and Al^18^F-NOTA-HER2-BCH (B) PET/CT imaging of the same patient with multiple metastases before and after 3 months anti-HER2 treatment. The arrows show lesions.

Figure 5 presents a case of a 57-year-old female newly diagnosed with metastatic breast cancer, who underwent initial lesion screening with Al^18^F-NOTA-HER2-BCH PET/CT. Following 3 months of anti-HER2 therapy (docetaxel/trastuzumab/pertuzumab), a second imaging evaluation revealed the disappearance of the primary lesion and diffuse lung metastases, signifying a clinical CR.

## Discussion

Patients with advanced HER2-positive breast cancer typically face a poor prognosis and require continuous anti-HER2 therapy from an early stage to maximize survival benefits.^24^ The heterogeneity of HER2 expression in breast cancer necessitates re-biopsies of recurrent or metastatic sites to accurately identify the cancer subtype, as recommended by current guidelines.^25-28^ However, the practical application of re-biopsy in clinical settings is often constrained due to various factors. Numerous studies have highlighted the potential of HER2-targeted PET/CT imaging as a non-invasive alternative for evaluating HER2 expression, leveraging whole-body imaging capabilities over limited lesion biopsies.^29-32^

Preclinical and clinical studies have explored the use of molecular imaging based on HER2 antibodies, including trastuzumab and pertuzumab labeled with isotopes like ^64^Cu (half-life: 12.7 hours) and ^89^Zr (half-life: 78.4 hours), for PET/CT imaging in gastric and patients with breast cancer.^33-36^ Notably, the ZEPHIR trial’s outcomes demonstrated that ^89^Zr-labeled trastuzumab (HER2) PET/CT, whether used alone or in combination with early FDG PET/CT, could evaluate HER2 heterogeneity in HER2-positive breast cancer and aid in identifying lesions and patients unlikely to respond to T-DM1 therapy.^37^ However, the slow clearance of antibody-based radiotracers from the blood and normal organs prompts the investigation into small-frame HER2 radiotracers. ZHER2:342, a molecular single-chain structure composed of 58 amino acid residues forming 3 α-helices, exhibits specific binding to HER2.^16^ Recent advancements in the field are increasingly focusing on the use of radionuclide-labeled ZHER2:342 and its analogs for tumor molecular imaging. In our study, we used Al^18^F-NOTA-HER2-BCH for HER2-targeted PET/CT imaging.

Preclinical studies have demonstrated that HER2-affinity imaging agents accumulate preferentially in xenograft tumors exhibiting elevated levels of HER2 expression.^17^ Such agents have been used to identify metastases in breast cancer and to assess the efficacy of anti-HER2-targeted therapies, evidenced by a notable decrease in the uptake of HER2-affinity agents by xenograft tumors following treatment with trastuzumab.^38^ In our study, the SUVmax of lesions prior to anti-HER2 therapy (*n* = 23) were significantly higher than those observed post-therapy (*n* = 16) in HER2 IHC 3 + lesions (19.9, 95%CI: 15.7-25.3 vs 9.8, 95%CI: 5.6-14.7; *P* = .006), aligning with the above findings. Additionally, a significant positive correlation was identified between the SUVmax from HER2-targeted PET/CT imaging prior to therapy and the pathological HER2 status determined by IHC (*n* = 27) (*P* = .034). The SUVmax values of lesions with positive HER2 pathology before therapy were substantially higher compared to those with negative HER2 status (17.9 ± 13.2 vs 1.1 ± 0.3; *P* = .007). Prior research has corroborated this, indicating a significant positive association between the level of HER2 expression in tumors and tracer uptake.^32,39^ Consequently, HER2-targeted PET/CT imaging emerges as a promising tool for accurately reflecting HER2 status, offering critical insights particularly in scenarios where biopsy results are not available.

Our study elucidates the heterogeneity of HER2 expression across primary and metastatic sites, as well as among different metastases, using both pathology and HER2-targeted PET/CT imaging to demonstrate spatial interlesional heterogeneity. In our cohort, 22.9% of patients (8 out of 35) exhibited variation in HER2 expression between primary and metastatic sites upon pathological examination. Similarly, 26.7% of patients (8 out of 30) displayed differences in HER2 expression across various sites, as evidenced by SUVmax values in HER2-targeted PET/CT imaging. These findings are in line with prior studies, which reported that up to 34% of patients with breast cancer present with heterogeneous HER2 expression.^25-28^ Such phenotypic alterations significantly complicate the management of HER2-positive breast cancer, highlighting the inadequacy of systemic treatments predicated solely on the subtype of the primary tumor for achieving optimal efficacy. Consequently, there is a pressing need for re-biopsy to capture these dynamic changes.^8^ The deployment of noninvasive, precise, and repeatable methodologies for assessing HER2 expression levels could offer a viable solution to these clinical predicaments and challenges.

In a recent study, the combination of ^64^Cu-DOTA-trastuzumab PET imaging data with MRI and a mathematical model was used to predict patient-specific responses to neoadjuvant chemotherapy and HER2-targeted therapy.^40^ Furthermore, it has been documented that ^64^Cu-DOTA-trastuzumab PET/CT imaging can predict the efficacy of HER2-targeted treatments by quantifying HER2 expression in patients. This predictive capability aligns with observations that lesions expressing HER2 respond more favorably to anti-HER2 therapy compared to those that are HER2 negative.^41^ Our previous study focused on the utility of ^68^Ga-NOTA-MAL-MZHER2 PET imaging in evaluating therapeutic outcomes in patients with advanced gastric cancer, revealing that patients with high ^68^Ga-HER2 affibody uptake in lesions experienced prolonged PFS durations of 4-9 months, in contrast to 2-3 months for those with low uptake.^23^ In the current study, we investigated the relationship between the SUVmax obtained from HER2-targeted PET/CT imaging and tumor response, including therapeutic outcomes and PFS, during both neoadjuvant and first-line therapy settings. Our findings indicate a trend toward a more favorable response associated with higher pretreatment SUVmax values, although this did not achieve statistical significance, likely attributable to the limited sample size and the relatively brief follow-up duration.

Importantly, no adverse events related to the injection of HER2 PET tracers were reported among study participants, underscoring the feasibility and safety of HER2-targeted PET/CT as a method for assessing HER2 status in patients with breast cancer. This technique offers a noninvasive, whole-body approach for evaluating lesion HER2 status and facilitates the re-assessment of HER2 expression following disease recurrence or metastasis.

Hence, our findings suggest that Al^18^F-NOTA-HER2-BCH PET/CT imaging offers a viable alternative to conventional nonspecific radiotracers for the noninvasive assessment of HER2 expression in breast cancer lesions. The rarity of repeated biopsies on identical metastatic tumors has resulted in a gap in knowledge regarding the evolution of HER2 expression throughout treatment. Given that a significant proportion of metastatic breast cancer cases eventually manifest treatment resistance, sequential PET imaging could serve as an early indicator of suboptimal response to anti-HER2 therapy. Real-time quantitative analysis via Al^18^F-NOTA-HER2-BCH PET/CT provides a mechanism to monitor HER2 levels during anti-HER2 treatment, thus holding considerable potential for prognostic evaluation, therapeutic decision-making, and detection of treatment resistance in breast cancer.

Our study had several limitations, notably the relatively small sample size, which restricted the variability in HER2 status representation. Ethical and practical considerations precluded histopathological verification of each detected lesion, and a singular biopsy per patient constrained our ability to pathologically examine intra-patient heterogeneity. Additional studies are warranted to further investigate the relationship between therapeutic response and SUVmax changes in HER2-targeted PET/CT, enhancing the depth of our research in future phases.

## Conclusion

In conclusion, HER2-targeted PET/CT imaging enables the real-time, non-invasive, and quantitative detection of HER2 expression across different lesions in patients with breast cancer, effectively addressing challenges such as re-biopsy difficulties, tumor heterogeneity, and the comprehensive assessment of therapeutic efficacy in clinical settings. This innovative imaging modality promises to facilitate the evaluation of HER2 status prior to therapy and the monitoring of HER2 expression dynamics during treatment. Ultimately, this could empower oncologists to tailor and refine therapeutic strategies for patients with breast cancer, promoting personalized, and optimized care.

## Data Availability

All datasets used and/or analyzed are available from the corresponding author on reasonable request.
